# Outcome after total ankle replacement or ankle arthrodesis in end-stage ankle osteoarthritis on the basis of german-wide data: a retrospective comparative study over 10 years

**DOI:** 10.1186/s12891-024-07612-w

**Published:** 2024-06-25

**Authors:** Tanja Kostuj, Alexander Hönning, Wolfram Mittelmeier, Jürgen Malzahn, Mike H. Baums, Katrin Osmanski-Zenk

**Affiliations:** 1 Orthopädisch-Traumatologisches Zentrum, St. Marien-Hospital Hamm, Nassauerstraße 13-19, 59065 Hamm, Germany; 2 Zentrum für Klinische Forschung, BG Klinikum Unfallkrankenhaus Berlin, Berlin, Germany; 3 Orthopädische Klinik und Poliklinik, Universitätsmedizin Rostock, Rostock, Germany; 4 AOK Bundesverband, Berlin, Germany; 5 Fachbereich Orthopädie, Katholisches Klinikum Ruhrgebiet Nord (KKRN), Unfallchirurgie und Sporttraumatologie, Dorsten, Germany

**Keywords:** Ankle arthrodesis, Total ankle replacement, Ankle fusion, Revision rate, Unplanned surgery

## Abstract

**Background:**

In symptomatic end-stage osteoarthritis of the ankle joint, total ankle replacement and ankle arthrodesis are the two primary surgical options for patients for whom conservative treatment fails. Published revision rates are often biased and difficult to compare. In this study, unplanned reoperation rates and revision rates were determined for both surgical interventions based on a large dataset, and risk factors for unplanned reoperations were identified.

**Methods:**

German-wide health data of the largest German health-care insurance carrier between 2001 and 2012 were retrospectively analyzed, and unplanned reoperation rates within 10 years were determined for index surgeries conducted in 2001 and 2002. Unplanned reoperation rates within 5 years for index surgeries conducted in 2001/2002 were compared to index surgeries conducted in 2006/2007. Multivariate logistic regression was used to identify risk factors for unplanned reoperations.

**Results:**

After ankle arthrodesis, 19% (95% confidence interval [CI], 16–22%) of 741 patients needed to undergo an unplanned reoperation within ten years. After total ankle replacement, the unplanned reoperation rate was 38% [95% CI, 29–48%] among 172 patients. For initial surgeries conducted at a later date, unplanned reoperation rates within five years were 21% [95% CI, 19–24%] for 1,168 ankle arthrodesis patients and 23% [95% CI, 19–28%] for 561 total ankle replacement patients. Significant risk factors for unplanned reoperations after ankle arthrodesis in the initial cohort were age < 50 years (odds ratio [OR] = 4.65 [95% CI 1.10;19.56]) and osteoporosis (OR = 3.72 [95% CI, 1.06;13.11]); after total ankle replacement, they were osteoporosis (OR = 2.96 [95% CI, 1.65;5.31]), Patient Clinical Complexity Level (PCCL) grade 3 (OR = 2.19 [95% CI, 1.19;4.03]), PCCL grade 4 (OR = 2.51 [95% CI, 1.22;5.17]) and diabetes mellitus (OR = 2.48 [95% CI, 1.33;4.66]). Kaplan-Meier analyses including 1,525 ankle arthrodesis patients and 644 total ankle replacement patients revealed an average unplanned reoperation-free time of approximately 17 years for both procedures.

**Conclusions:**

Similar revision rates and unplanned reoperation rates for both procedures in the later-date cohort can likely be attributed to a learning curve for surgeons as well as advances in implant design. This analysis of billing health insurance data supports an increase in total ankle replacement surgeries.

**Supplementary Information:**

The online version contains supplementary material available at 10.1186/s12891-024-07612-w.

## Background

In patients with symptomatic end-stage osteoarthritis of the ankle joint for whom conservative therapeutic approaches fail, ankle arthrodesis has long been considered the gold standard for treatment [1–3]. With more recent advances in implant design, particularly the development of a cementless implantation technique, the long-term implant survival in total ankle replacement has improved significantly in association with a reduction in complication and revision rates [1, 4–7]. Therefore, total ankle replacement is now regarded as an equivalent treatment alternative for numerous indications in patients with end-stage osteoarthritis [8, 9]. However, the 3,000–4,000 ankle arthrodesis surgeries performed annually in Germany far exceeds the number of total ankle replacement surgeries, which is approximately 1,000 per year [10].

Robust evidence on unplanned reoperation rates and revision rates of these two surgical treatments is largely lacking because published studies only examine patient outcomes after application of one of the two procedures. A recently published prospective, randomized, multicenter study directly comparing patient outcomes of both therapies included only a 1-year follow-up [11]. Another multicenter randomized controlled trial started by the research group led by M. Glazebrook [12] is ongoing. In terms of functional outcomes and postoperative pain, total ankle replacement showed better results in the majority of studies [6, 13–17]. However, for complication and revision rates, there were sometimes major differences between studies and contradictory results [5, 8, 13, 18–27]. For example, longer implant survival was reported in specialized centers [15, 28–30] than in national registries [31–35] or meta-analyses [8, 14, 19, 21, 36], resulting in 10-year implant survival times ranging from 56.5% [37] to 92% [38]. Thus, an objective comparison of unplanned reoperation rates and revision rates of both procedures is not possible based on currently published data. In this retrospective analysis, pseudonymized German-wide insurance data of the Allgemeine Ortskrankenkasse (AOK) on ankle arthrodesis and total ankle replacement were evaluated.

This study aimed to complement the current state of research with findings regarding unplanned reoperation rates and revision rates after ankle arthrodesis and total ankle replacement. Identification of risk factors for unplanned reoperations of both procedures should provide guidance on whether these correspond to indications and contraindications described in the literature. Another focus of the analysis was to compare an earlier-date patient population with initial procedures in 2001 and 2002 with a later-date population with initial procedures in 2006 and 2007. This comparison should help to evaluate changes in unplanned reoperation rates and revision rates based on a possible learning curve of the surgeons performing total ankle replacements as well as technical innovations in implants. In addition, sensitivity analyses were performed to assess potential confounding of the results by lost-to-follow-up, as caused by patients switching health insurance or dying.

## Methods

### Study design

This retrospective analysis on insurance data was approved by the responsible ethics committee (Ethics Committee at the Medical faculty of the University of Rostock, Germany, A 2020-0030) in January 2020. The pseudonymized dataset provided by the AOK insurance included all primary ankle arthrodesis and total ankle replacement interventions performed in Germany between January 2001 and December 2012, including data on follow-up procedures, complications and patient characteristics. Import and synthesis of the password-encrypted Excel-compatible files as well as case selection and creation of the analysis sets was performed using SPSS^®^ version 28 (IBM^®^, Armonk, NY, USA).

### Case Selection and Analysis Sets

Patients who underwent ankle arthrodesis or total ankle replacement between January 1, 2001, and December 31, 2002 and between January 1, 2006 and December 31, 2007, were followed-up for 5 and/or 10 years. Patients with open fractures of the distal end of the tibia, malleoli, or talus (grades II and III) were excluded from the analysis due to the increased risk of infection, potentially leading to biased results.

The complete case set comprised only patients consistently insured by AOK and for whom data were available for at least 5 or 10 years since the initial surgery. The non-complete case set also included patients who switched health insurance or died within 5 or 10 years.

**Fig. 1:**
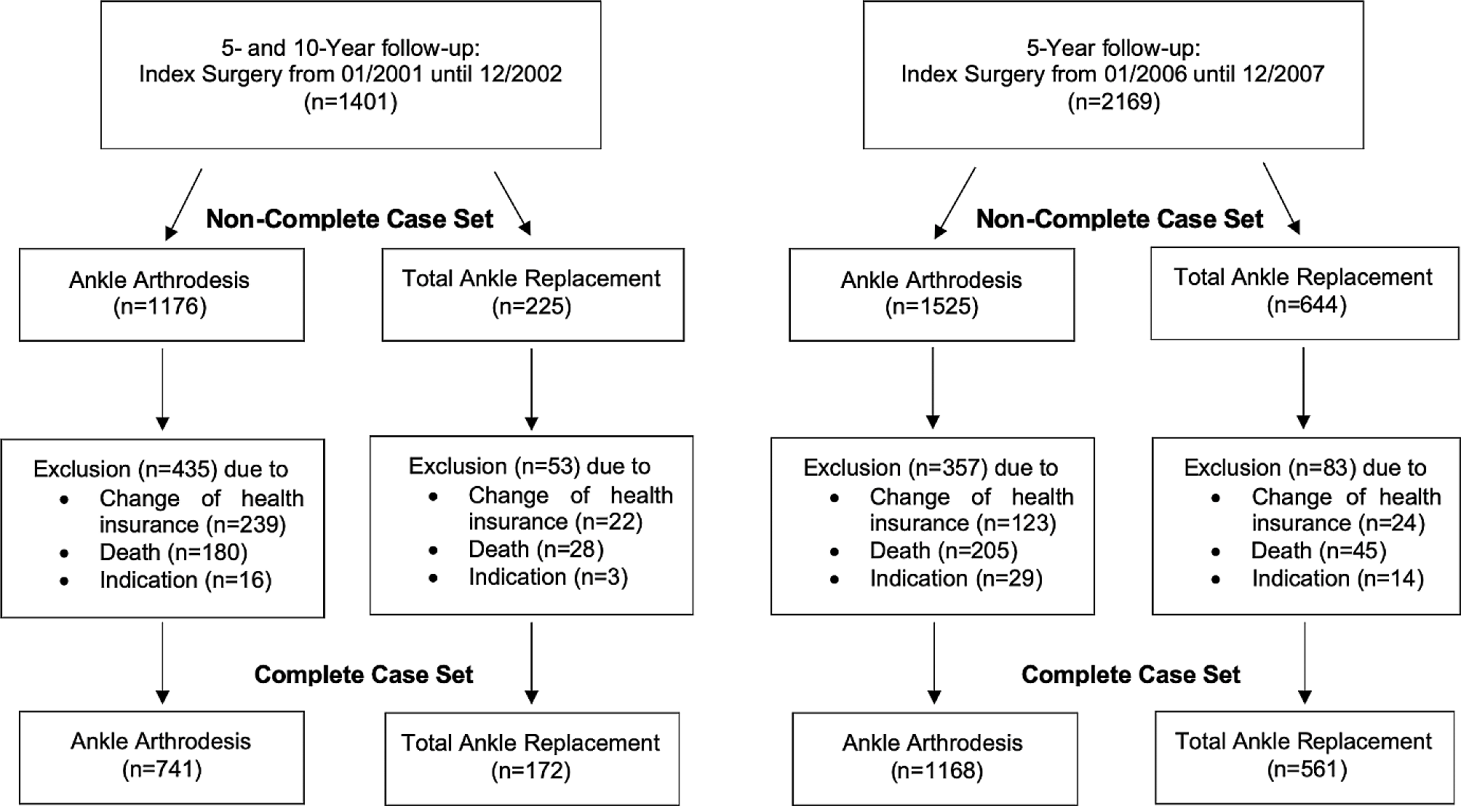
Flow chart of the analysis sets

Figure 1 shows a flow chart of all analysis sets including the numbers of drop-outs with their respective reasons.

### Data

The AOK data set used in this analysis consists of billing data for inpatient hospital treatment across all 16 German states. Due to the AOK’s coverage of more than one third of all statutory insured patients in Germany across most federal states, this study ensured a high representativeness of the sample for the surgical treatment of ankle joint osteoarthritis. Indeed, a uniform recording of initial surgeries and follow-up procedures based on one patient collective enables a fair comparison with regard to unplanned reoperation rates and revision rates that realistically reflect the care situation in Germany. Treatment information, date of admission to hospital, date of surgery, date of discharge, Diagnosis Related Group (DRG) data and Patient Clinical Complexity Level (PCCL) are provided as treatment information. PCCL indicates the effect of complications and comorbidities in a patient and ranges from 0 (no complication or comorbidity) to 4 (very severe complication or comorbidity) and was shown to be highly correlated with patient outcome after total hip and total knee arthroplasties [39]. The type of surgery performed is specified precisely by Operationen- und Prozedurenschlüssel (OPS = operation and procedure code) [40]. Ankle arthrodesis was identified via OPS code 5-808.7; total ankle replacement was identified by OPS code 5-826.0. International Statistical Classification of Diseases and Related Health Problems (ICD) codes provide information on the presence of diabetes mellitus, osteoporosis and obesity as well as complications potentially associated with any of the surgeries [41]. ICD coding is also available for the type of osteoarthritis, but in most cases it was coded as unspecified osteoarthritis. Master data of the patients include date of birth and, if deceased, date of death as well as sex, insurance status and place of residence.

The AOK data set did not contain any information on the postoperative protocols applied or the implants used. However, during the period under review, the vast majority of total ankle replacements in Germany involved the use of uncemented three component implants such as the STAR™, the HINTEGRA^®^ or the SALTO^®^ which had to be approved as medical devices by notified bodies in Germany. There was no selection done, neither for the arthrodesis implants nor for the total ankle replacements. Fusions were done by using screws, plates and nails.

### Definition of unplanned reoperation and revision

Unplanned reoperation is defined as any unplanned follow-up procedures after an initial ankle arthrodesis or total ankle replacement presumably related to the initial procedure (Table 1). Revision surgeries only comprise rearthrodeses, osteosyntheses, conversions of an arthrodesis to an total ankle replacement and vice versa, and revision of any prosthetic component and removal of the total ankle replacement. In addition, operations on the skin and subcutaneous tissue of the ankle joint were only counted as unplanned reoperations if they were conducted within three months of the initial procedure, as we considered a causal relationship with the index surgery unlikely after this time window. If osteosyntheses after an initial ankle arthrodesis were coded with fractures (i.e., OPS codes S82, S92, or S93) at the time of surgery, no relationship to the arthrodesis procedure was assumed, and the surgery was also not defined as an unplanned reoperation or revision.

**Table 1:** Definition of unplanned reoperations and revisions

Unplanned Reoperation		Revision	
Ankle Arthrodesis	Total Ankle Replacement	Ankle Arthrodesis	Total Ankle Replacement
Rearthrodesis	Conversion to arthrodesis	Rearthrodesis	Conversion to arthrodesis
Osteosynthesis of bone	Removal of total ankle replacement	Osteosynthesis of bone	Removal of total ankle replacement
Conversion to total ankle replacement	Revision of any prosthetic component including liner exchange	Conversion to total ankle replacement	Revision of any prosthetic component including liner exchange
	Exposure of total ankle replacement without revision		
Operations on skin and subcutaneous tissue (OPS codes 5-89x to 5-92x)	Operations on skin and subcutaneous tissue (OPS codes 5-89x to 5-92x)		

### Statistical analyses

Unplanned reoperation rates and revision rates were calculated as cumulative incidence rates for the complete case set, describing the probability that an event occurred in a patient for the first time within a predefined period of time (in this study, 5 or 10 years). The cumulative incidence rate can be between 0 and 1. Thus, for a short observation period, the cumulative incidence is usually close to 0 and rises with increasing duration. Cumulative incidence rates are reported with associated 95% Clopper-Pearson confidence intervals (CI).

Patients without complete documentation who switched health insurance or died within the observation period (“non-completers”) were also considered in the Kaplan-Meier analysis. Thus, both the time until occurrence of the unplanned reoperation and the time until censoring were taken into account in the estimator. After each unplanned reoperation, the probability of not suffering an unplanned reoperation decreases and the Kaplan-Meier curve drops. The reoperation-free survival of ankle arthrodesis group and total ankle replacement group is compared using the logrank test. It tests the null hypothesis that there is no difference in the probability of unplanned reoperations between the two groups.

Logistic regression analyses were performed to investigate the influence of different variables on risk of an unplanned reoperation separately for both surgical procedures. The variables age group (0–17 years, 18–49 years, 50–69 years, >=70 years), sex (male, female), PCCL (0, 1, 2, 3 or 4), diabetes mellitus (yes/no), obesity (yes/no; according to the WHO definition, the threshold for obesity is defined here as a body mass index [BMI] of > = 30) and osteoporosis (yes/no), which are described in the literature as potential risk factors, were included as predictors in the regression model with the response “number of patients with at least one unplanned reoperation”. The quality of the model was assessed based on Cohen’s effect size f^2^ [42]. The following reference values were used to help classify the size of the effect: f^2^ = 0.02 ~ weak effect, f^2^ = 0.15 ~ medium effect, f^2^ = 0.35 ~ strong effect.

The significance of odds ratios (OR) was examined using the 95% CI of ORs. If the 95% CI did not include 1, a significant influence of the predictor or the level of the respective predictor was assumed. To identify statistically significant differences between groups of patient who underwent an ankle arthrodesis or a total ankle replacement, selected demographic variables and comorbidities were compared at the time of the index surgery. In the case of the continuously measured variable age, this was achieved using a t-test for independent samples. Variables of categorical scale level [i.e., sex, PCCL, diabetes mellitus (yes/no), obesity (yes/no) and osteoporosis (yes/no)] were tested for significant differences at a significance level of 0.05 by Pearson’s chi-square test or, in the case of cell frequency < 5, by Fisher’s exact test.

## Results

### Baseline characteristics

In the 10-year follow-up complete case set, the 913 included patients were on average 54.6 (+- 14.9) years of age, the sex distribution was homogenous, and 19% had obesity. Additionally, 69.4% had PCCL severity grade 0; diabetes mellitus was present in 10.3% of the patients and osteoporosis in 15.3%.

Significant differences between patients with ankle arthrodesis and total ankle replacement were found for age (t(311.7))=-4.30, p = < 0.001) and sex (χ^2^ [1] = 6.46, *p* = 0.011). With regard to comorbidities, no significant differences between groups were detected, although patients in the total ankle replacement group had slightly fewer comorbidities and a lower overall PCCL severity (Table 2).

**Table 2:** Patients’ baseline characteristics

Variable	Ankle Arthrodesis (*N* = 741)	Total Ankle Replacement (*N* = 172)	Total (*N* = 913)
Age, Average ± SD [Range]**	53.8 ± 15.3 [0–82]	58.4 ± 12.1 [21–83]	54.6 ± 14.9 [0–83]
Age Group, n (%)**			
0–17	19 (2.6)	0 (0.0)	19 (2.1)
18–49	245 (33.1)	39 (22.7)	284 (31.1)
50–69	382 (51.6)	106 (61.6)	488 (53.5)
>=70	95 (12.8)	27 (15.7)	122 (13.4)
Sex, n (%)*			
Female	334 (45.1)	96 (55.8)	430 (47.1)
Male	407 (54.9)	76 (44.2)	483 (52.9)
PCCL severity grade, n (%)			
0	499 (67.3)	135 (78.5)	634 (69.4)
1	2 (0.3)	0 (0.0)	2 (0.2)
2	62 (8.4)	9 (5.2)	71 (7.8)
3	100 (13.5)	17 (9.9)	117 (12.8)
4	66 (8.9)	9 (5.2)	75 (8.2)
Diabetes mellitus, n (%)	83 (11.2)	11 (6.4)	94 (10.3)
Osteoporosis, n (%)	120 (16.2)	20 (11.6)	140 (15.3)
Obesity, n (%)	142 (19.2)	31 (18.0)	173 (19.0)

SD = Standard Deviation, n = Number of Patients, %=Proportion of Patients, PCCL = Patient Clinical Complexity Level, *p < 0.05, **p < 0.01

In the 5-year follow-up complete case set, patients were older compared to the 10-year follow-up complete case set with a mean average of 58.6 (± 14.1) years for ankle arthrodesis and a mean average of 59.7 (± 12.2) years for total ankle replacement. Moreover, PCCL severity was significantly higher with 17.0% of patients having PCCL 3 grade and 17.2% of patients having PCCL 4 grade for ankle arthrodesis as well as 17.1% of patients having PCCL 3 grade and 8.9% of patients having PCCL 4 grade for total ankle replacement. The proportion of patients with diabetes mellitus was also considerably higher with 18.2% in the ankle arthrodesis group and 11.2% in the total ankle replacement group (see Appendix Table 1).

### Number of index surgeries

**Fig. 2:**
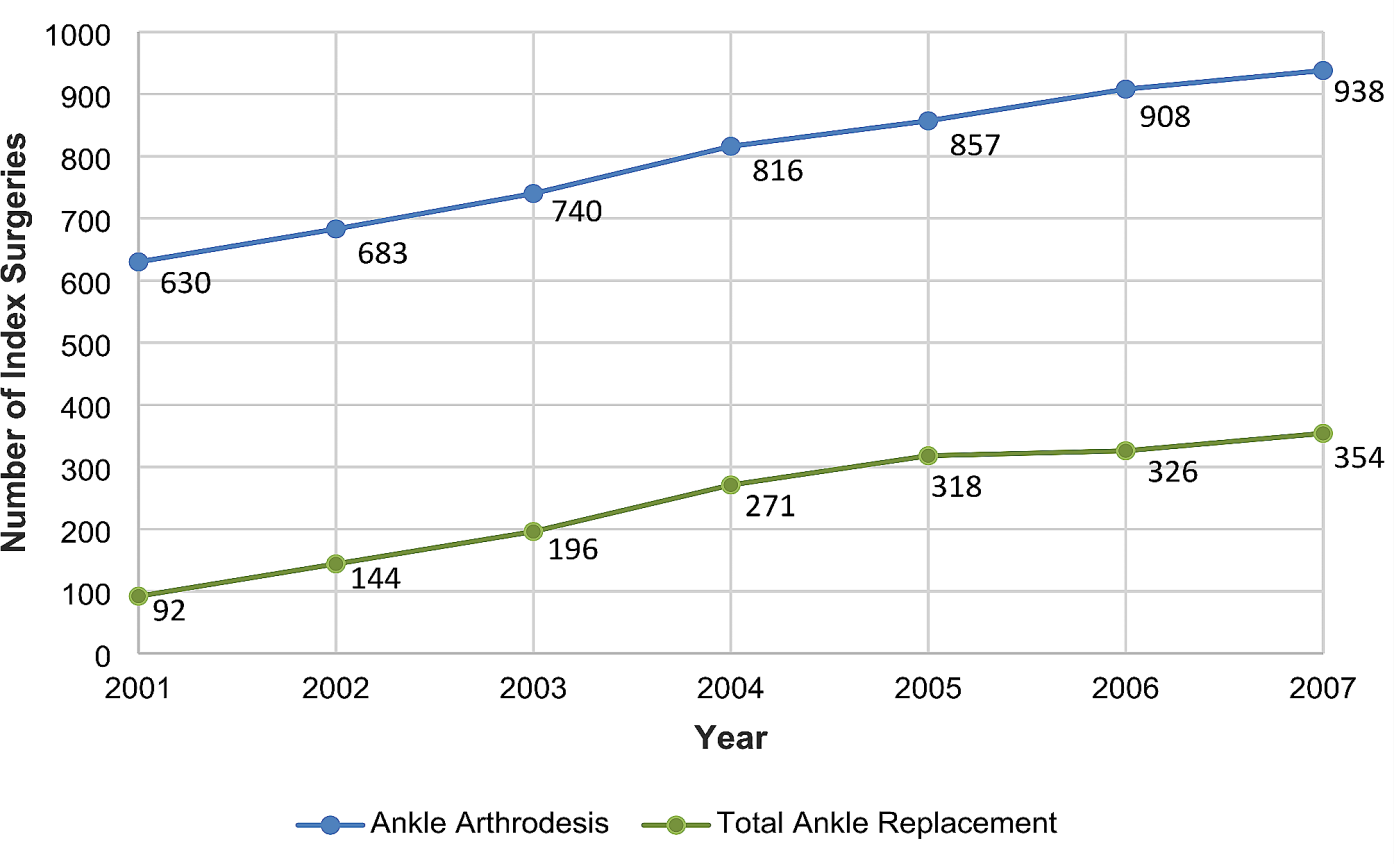
Number of Index Surgeries (2001–2007)

The number of both surgical interventions increased between 2001 and 2007. However, while the number of ankle arthrodesis procedures increased only approximately 1.5-fold, from 630 to 938, the number of total ankle replacement procedures increased approximately 3.8-fold, from 92 to 354 surgeries. Compared with the first year of the observation period, namely, 2001, the ratio of total ankle replacements to ankle arthrodeses increased from 1:7 to less than 1:3 in 2007 (Fig. 2). The total number of AOK insured patients decreased from 26,405,444 to 24,490,589 during the same period of time.

### Cumulative incidence of unplanned reoperations – influence of missing values

Of the patients in the 10-year follow-up complete case set, 741 underwent ankle arthrodesis and 172 total ankle replacement in 2001 or 2002.

After ankle arthrodesis, 19% [95% CI, 16–22%] of patients had at least one follow-up procedure within ten years, primarily due to rearthrodesis, osteosynthesis of the bone or debridement of the skin (within 90 days post-operation). After ankle replacement, the proportion of patients with at least one follow-up procedure within ten years was approximately 38% [95% CI, 29–48%]. The most common types of unplanned reoperations were conversion to ankle arthrodesis and removal or replacement of the total ankle replacement. Within ten years, 23 (3.1%) cases of transtibial amputations after ankle arthrodesis occurred, and only 2 (1.2%) after total ankle replacement.

When including non-completers, the number of patients at risk increased from 741 to 1,176 in the ankle arthrodesis group and from 172 to 225 in the total ankle replacement group compared with the complete case set. Of the 435 (37%) non-completers, 186 patients (43%) died within 10 years after ankle arthrodesis, and 249 (57%) patients changed health insurance. Of the 53 (24%) non-completers in the total ankle replacement group, 30 (58%) patients died, and 23 (42%) patients changed health insurance. While 23% of the non-completers underwent an unplanned reoperation after ankle arthrodesis, this proportion was only 21% after total ankle replacement.

Accordingly, the differences in the incidences of unplanned reoperations between the two collectives became somewhat smaller in the non-complete case set, with a cumulative incidence of 18% [95% CI, 15–20%] after ankle arthrodesis and 33% [95% CI, 26–42%] after total ankle replacement (Table 3).

**Table 3:** Unplanned reoperations within 10 years – complete cases vs. non-complete cases

	Complete Case Set (*N* = 913)		Non-Complete Case Set (*N* = 1401)	
Ankle Arthrodesis	Patients (*N* = 741), *n* (%)*	Surgeries n**	Patients (*N* = 1176), *n* (%)*	Surgeries n**
Rearthrodesis	100 (14)	144	147 (12)	199
Osteosynthesis of the bone	55 (7)	68	76 (6)	90
Operations on the skin and subcutaneous tissue	24 (3)	40	39 (3)	62
Conversion to total ankle replacement	8 (1)	8	9 (1)	9
Sum of patients with unplanned reoperations/ sum of surgeries	140 (19***)	260	206 (18***)	360
**Total Ankle Replacement**	**Patients (*N* = 172)**, n (%)*	**Surgeries** n**	**Patients (*N* = 225)**, n (%)*	**Surgeries** n**
Conversion to ankle arthrodesis	39 (23)	70	43 (19)	76
Removal of total ankle replacement	34 (20)	34	38 (17)	38
Revision of total ankle replacement or individual components	25 (14)	42	26 (11)	41
Exposure of total ankle replacement without replacement	11 (6)	13	15 (7)	19
Operations on the skin and subcutaneous tissue	10 (6)	12	8 (4)	10
Sum of patients with unplanned reoperations/sum of surgeries	65 (38***)	171	75 (33***)	184

*=Number (Proportion) of patients with at least one event, **=Number of surgeries

***=Patients with different types of surgeries are only counted once in the sum - for this reason, the sum of patients does not correspond to the sum of number of patients in the subcategories

### Cumulative incidence of unplanned reoperations – influence of the index surgery year

To determine whether the incidences of unplanned reoperations differed over the course of the observation period, the incidences of unplanned reoperations were considered at an earlier time frame (index surgery from 01/2001 to 12/2002) and at a later time frame (index surgery from 01/2006 to 12/2007). In the earlier-date cohort, the incidence after ankle arthrodesis (i.e., 17% [95% CI 14–20%]) was slightly lower than the incidence after total ankle replacement (i.e., 25% [95% CI 18–34%]). In the later-date cohort, the incidences were in a similar range for both procedures, with a proportion of 21% [95% CI 19–24%] after ankle arthrodesis and 23% [95% CI 19–28%] after total ankle replacement. As in the 10-year follow-up, unplanned reoperations after an initial ankle arthrodesis were primarily undertaken due to rearthrodesis, osteosynthesis of the bone or debridement of the skin (within 90 days post-operation); after initial total ankle replacement, unplanned reoperations were mainly conducted due to conversion of the total ankle replacement to an ankle arthrodesis and removal or replacement of the total ankle replacement (Table 4).

**Table 4:** Unplanned reoperations within 5 years – early cohort vs. late cohort

	Index Surgery in 01/2001-12/2002 (*N* = 913)		Index Surgery in 01/2006-12/2007 Set (*N* = 1729)	
Ankle Arthrodesis	Patients (*N* = 741), *n* (%)*	Surgeries n**	Patients (*N* = 1168), *n* (%)*	Surgeries n**
Rearthrodesis	88 (12)	105	161 (14)	290
Osteosynthesis of the bone	42 (6)	46	129 (11)	161
Operations on the skin and subcutaneous tissue	24 (3)	41	67 (6)	114
Conversion to total ankle replacement	4 (1)	4	4 (0.3)	4
Sum of patients with unplanned reoperations/sum of surgeries	122 (17***)	196	246 (21***)	569
**Total Ankle Replacement**	**Patients (*N* = 172)**, n (%)*	**Surgeries** n**	**Patients (*N* = 561)**, n (%)*	**Surgeries** n**
Conversion to ankle arthrodesis	23 (13)	26	60 (11)	70
Removal of total ankle replacement	20 (12)	20	58 (10)	58
Revision of total ankle replacement or individual components	11 (6)	14	58 (10)	67
Exposure of total ankle replacement without replacement	7 (4)	8	26 (5)	28
Operations on the skin and subcutaneous tissue	10 (6)	12	23 (4)	24
Sum of patients with unplanned reoperations/sum of surgeries	43 (25***)	80	130 (23***)	247

*=Number (Proportion) of patients with at least one event, **=Number of surgeries

***=Patients with different types of surgeries are only counted once in the sum - for this reason, the sum of patients does not correspond to the sum of number of patients in the subcategories

### Unplanned reoperation-free time – Kaplan-Meier analyses

**Fig. 3:**
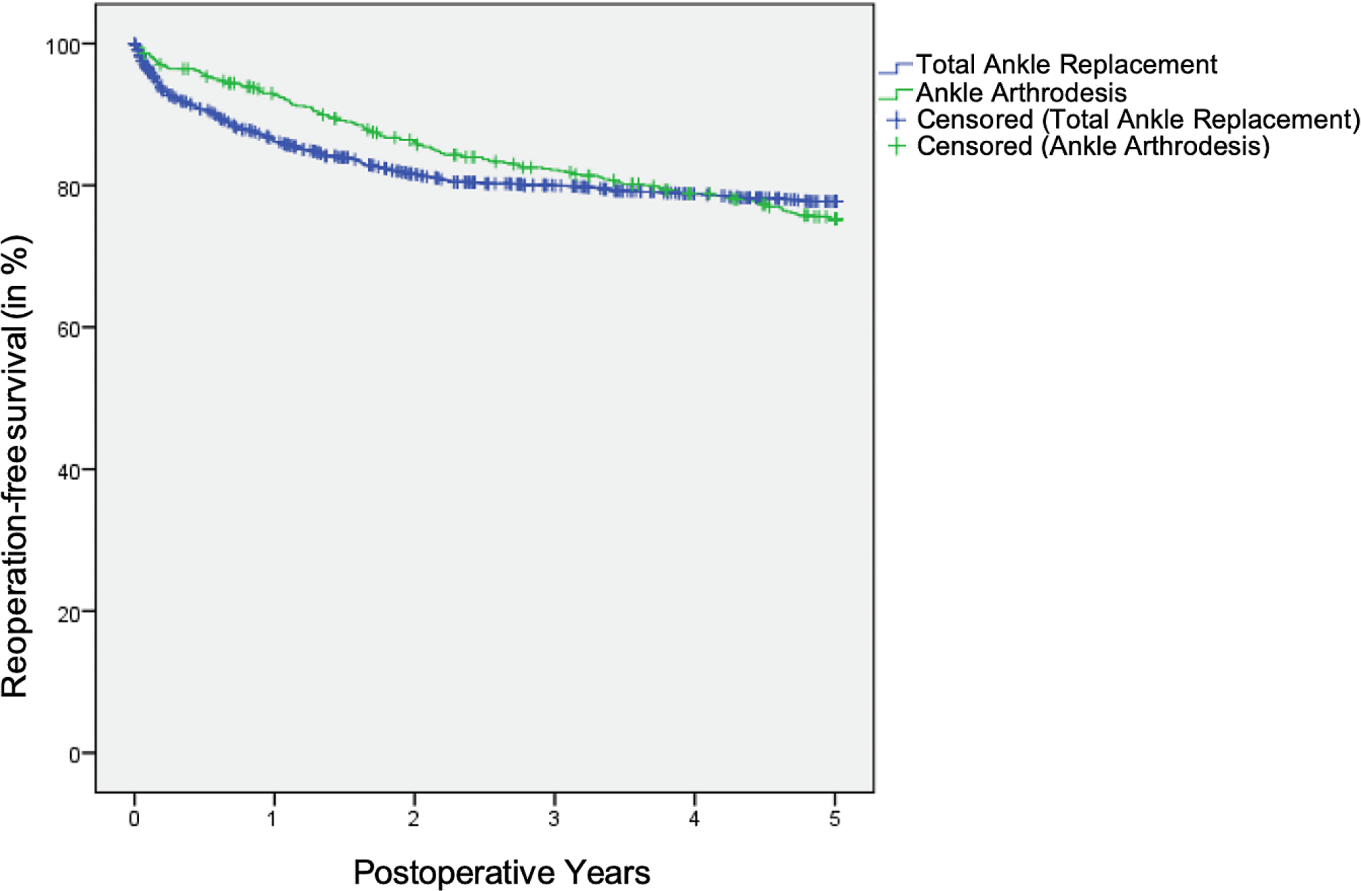
Kaplan meier curves – time to unplanned reoperation. After total ankle replacement, unplanned reoperations were approximately evenly distributed over the observation period. After ankle arthrodesis, events occurred significantly more frequently at the beginning of the period. With increasing observation time, the curves converge, and finally cross toward the end of the observation period

While previous calculations only took into account whether and how many unplanned reoperations occurred in a predefined period, the Kaplan-Meier method curves (Fig. 3) also showed when these events occurred between the index surgery in 2006 or 2007 and the end of the observation period for 644 patients who underwent total ankle replacement and 1,525 patients who underwent ankle arthrodesis. Five years after the index surgery the Kaplan-Meier estimators were at a similar level with a proportion of patients without an unplanned reoperation ranging from 75% (i.e., total ankle replacement) to 78% (i.e., ankle arthrodesis). Based on the Kaplan-Meier survival analysis, the average unplanned reoperation-free time that a patient could expect until the index surgery was estimated at 17.41 years [95% CI 16.96 to 17.87] after ankle arthrodesis and at 17.09 years [95% CI 16.44 to 17.76] after total ankle replacement. No significant difference in reoperation-free survival between ankle arthrodesis group and total ankle replacement group was found (χ2 = 0.16, *p* = 0.693).

### Identification of risk factors for unplanned reoperation rates

In two logistic regression analyses, separately for ankle arthrodesis patients and total ankle replacement patients, the parameters sex, age, PCCL, diabetes, obesity, and osteoporosis were tested for significant influence on the response “number of patients with at least one unplanned reoperation”.

For both regression models, a significant chi-square test revealed a relevant explanatory contribution compared to the modal prediction. In terms of model accuracy, the total ankle arthrodesis model had a Nagelkerke’s R-square value of 0.14, while the total ankle replacement model had a value of 0.17. When converted to Cohen’s effect size, the models achieved an effect of 0.40 and 0.45, respectively, which corresponds to a strong effect (> 0.35).

After ankle arthrodesis, the presence of osteoporosis (OR = 2.96 [95% CI, 1.65;5.31]), the presence of diabetes mellitus (OR = 2.48 [95% CI, 1.33;4.66]), PCCL severity level 3 (OR = 2.19 [95% CI, 1.19;4.03]) and PCCL severity level 4 (OR = 2.51 [95% CI, 1.22;5.17]) were identified as significant risk factors at a significance level of 5%. After total ankle replacement, only age < 50 (OR = 4.65 [95% CI, 1.10;19.56]) and the presence of osteoporosis (OR = 3.72 [95% CI, 1.06;13.11]) were significant influencing variables in the model (Table 5).

**Table 5:** Logistic regression models assessing factors associated with the occurrence of an unplanned reoperation (10-year follow-up)

Procedure	Covariates	Regression Coefficient	p-value	Odds Ratio	95% CI	
					Lower	Upper
Ankle Arthrodesis (*N* = 741)	Sex: Female	Reference				
	Sex: Male	0.345	0.171	1.411	0.861	2.315
	Age: 0–17 Years	-1.354	0.999	0.258	0.000	.
	Age: 18–49 Years	0.523	0.221	1.688	0.729	3.910
	Age: 50–69 Years	0.522	0.212	1.685	0.742	3.826
	Age: >=70 Years	Reference				
	PCCL Grade 0	Reference				
	PCCL Grade 1	-20.61	1.000	0.000	0.000	0.000
	PCCL Grade 2	0.387	0.361	1.472	0.639	3.391
	PCCL Grade 3*	0.782	0.012	2.185	1.185	4.030
	PCCL Grade 4*	0.922	0.012	2.514	1.222	5.173
	Diabetes mellitus: Yes**	0.910	0.005	2.484	1.325	4.656
	Diabetes mellitus: No	Reference				
	Osteoporosis: Yes***	1.086	0.000	2.961	1.651	5.311
	Osteoporosis: No	Reference				
	Obesity: Yes	0.506	0.098	1.659	0.910	3.024
	Obesity: No	Reference				
Total Ankle Replacement (*N* = 172)	Sex: Female	Reference				
	Sex: Male	0.632	0.115	1.882	0.857	4.132
	Age: 0–17 Years	---				
	Age: 18–49 Years*	1.536	0.036	4.647	1.104	19.559
	Age: 50–69 Years	0.829	0.195	2.290	0.653	8.034
	Age: >=70 Years	Reference				
	PCCL Grade 0					
	PCCL Grade 1					
	PCCL Grade 2	0.990	0.258	2.691	0.483	14.982
	PCCL Grade 3	0.663	0.355	1.940	0.474	7.948
	PCCL Grade 4	1.566	0.129	4.787	0.632	36.230
	Diabetes mellitus: Yes	− 0.017	0.983	0.983	0.196	4.920
	Diabetes mellitus: No	Reference				
	Osteoporosis: Yes*	1.313	0.041	3.719	1.055	13.110
	Osteoporosis: No	Reference				
	Obesity: Yes	0.864	0.082	2.373	0.896	6.283
	Obesity: No	Reference				

*p < 0.05, **p < 0.01, ***p < 0.001, CI = Confidence Interval, PCCL = Patient Clinical Complexity Level

The probability of unplanned reoperations in Table 5 is supplemented by reoperation rates stratified by age groups and sex for the 10-year follow-up cohort as well as the early and late 5-year follow-up cohorts (see Appendix Table 2). The age groups 18–49 years and 50–69 years predominantly show the highest proportion of reoperations for both surgical procedures, while the lowest proportion is observed for the age group > = 70 years. Due to the low total number of cases in the age group 0–17 years, this stratum does not allow any reliable conclusions to be drawn. In terms of sex, men had more frequently unplanned reoperations compared to women across both groups.

The presentation of rates of unplanned reoperations (see Appendix Table 3) by the type of osteoarthritis is impaired by the fact that only around 40% of all patients could be assigned to an osteoarthritis type due to a lack of ICD specification. After ankle arthrodesis, the proportion of unplanned reoperations in patients with primary osteoarthritis (ICD-10 M19.07) is higher (i.e., 27%) than after total ankle replacement (i.e., 21%). The opposite trend can be observed for secondary osteoarthritis (ICD-10 M19.27) with a proportion of unplanned reoperations of 20% after ankle arthrodesis vs. 37% after total ankle replacement and for post-traumatic osteoarthritis (ICD-10 M19.17) with a proportion of unplanned reoperations of 16% after ankle arthrodesis vs. 30% after total ankle replacement.

## Discussion

This retrospective analysis on insurance data showed that incidences of unplanned reoperation rates and revision rates after total ankle replacement were higher in the earlier study period and converged between the two procedures in the later study period. Based on evaluation of the literature, possible reasons for this will be discussed, and the results will be compared with findings from current studies.

### Differences between patient cohorts at baseline

The observed differences in total ankle replacement and ankle arthrodesis patient groups are largely consistent with the indications and contraindications of both procedures described in the relevant literature. For example, higher age and lower body mass index (BMI) in the total ankle replacement collective compared with the ankle arthrodesis collective is described as an indication for ankle replacement, in addition to low physical stress and malalignment [43, 44]. Furthermore, two literature reviews found an association between younger age (i.e., < 45 years and < 50 years) and a higher risk of total ankle replacement failure/unplanned reoperation in the majority of studies [1, 9]. In a clinical study of 684 patients, a 3.84 times higher odds ratio for implant failure was calculated for patients younger than 70 years compared to those older than 70 years [45].

Relative contraindications, especially for total ankle replacement, include the presence of osteoporosis and diabetes mellitus, in addition to poor bone quality and nicotine use [46], which is reflected by the less frequent occurrence of osteoporosis and diabetes mellitus among total ankle replacement patients in our data. However, an extensive review of the literature also identified these parameters as risk factors for complications after ankle arthrodesis [47].

The preference for ankle arthrodesis over total ankle replacement among men in our sample is also in line with current recommendations [18], as men are on average probably exposed to higher physical stress than women in the occupational context and are therefore also more frequently predisposed to ankle arthrodesis.

Differences in the incidence of unplanned reoperation rates and revision rates between the earlier study period (initial surgery in 2001 or 2002) and the later study period (initial surgery in 2006 or 2007) may originate from a heterogeneous sample composition of the ankle arthrodesis population in the two cohorts. It can be assumed that the increase in the number of cases of ankle arthrodeses and total ankle replacements in the period from 2001 to 2007 (Fig. 2) was accompanied by an expanded indication for surgery, which resulted in interventions in patients who were at higher risk for reoperations. As a result, patients in the later-date ankle arthrodesis population were on average older (58.6 years), had a significantly higher overall PCCL severity, and were significantly more likely to have diabetes mellitus (18.2% compared to 11.2%) compared with the 2001/2002 intervention period. These developments in subject characteristics were also evident in the total ankle replacement population (see Table 2 and Appendix Table 1).

### Unplanned reoperation rates and revision rates after total ankle replacement and ankle arthrodesis

In order to obtain a holistic picture of the rates of unplanned reoperations and revisions determined in this analysis, two important factors must be taken into account:

1. change of health insurance or death of the patient (i.e., non-completers) and.
2. year of index surgery.

With a proportion of 37% after ankle arthrodesis and 24% after total ankle replacement, a considerable amount of the study population dropped out of the 10-year complete case analysis population. However, these exclusions can lead to biased results and over- or underestimates of the calculated rates [48, 49] which is why we performed a sensitivity analysis based on the non-complete case set.

The proportion of patients who changed their health insurance company was about twice as high in the ankle arthrodesis group (21.2%) as in the total ankle replacement group (10.2%), which is probably due to the lower average age of the arthrodesis collective, as younger patients generally change health insurance more frequently [50]. The inclusion of non-completers in the analysis resulted in an approximation of the unplanned reoperation rates between total ankle replacement and ankle arthrodesis groups which suggests a bias in the complete case analysis due to right censored data [51].

Separate analyses of the two 5-year periods revealed that unplanned reoperations after ankle arthrodesis occurred more frequently within 5 years after index surgeries in 2006/2007 (in 21% of cases) than in the 5-year period after index surgery in 2001/2002 (in 17% of cases). In contrast, the cumulative incidence of unplanned reoperations after total ankle replacement was slightly lower in the later period (in 23% of cases) than in the earlier period (in 25% of cases). The main reason for the increase in unplanned reoperations after ankle arthrodesis is probably a higher initial risk of complications due to a higher proportion of comorbidities in the patient cohort (compare previous section). On the other hand, in the case of total ankle replacement, a greater surgeon experience with implants used may have offset the increase in unplanned reoperations caused by a broader indication for surgery. The 1990s saw decisive technical developments in total ankle replacement with the implementation of a cementless implantation technique and the introduction of the three-component system with a movable sliding core, which, after a certain trial phase, gradually replaced the previously used models and established itself as the standard [1, 3, 4]. The learning curve for surgeons associated with the use of new types of implants is generally described as rather flat in total ankle replacement [7, 46, 52] and is likely to be ongoing in many cases during the period analyzed. Therefore, a large number of operations are required for an optimal treatment result [53, 54]. The emergence of new providers for total ankle replacement in the 2000s also led to an initial phase during which specialist knowledge and experience had to be acquired step by step [52]. Comparing the outcome of routinely performed ankle arthrodesis procedures with the outcome of total ankle replacement presumably in the midst of a learning curve must therefore be interpreted with caution to avoid drawing biased conclusions.

When comparing our estimated rates with published data, it must be considered that observed differences are to some extent due to heterogeneous study designs, study populations, or analysis strategies [2].

The few comparative studies on outcome after ankle arthrodesis or total ankle replacement included 2 meta-analyses [8, 55], one multicenter study [6], and one single center study [56], which reported rather different unplanned reoperation and revision rates. Based on comparative studies, slightly lower unplanned reoperation and/or revision rates after total ankle replacement are reported in the majority of studies [6, 8, 55], though a better outcome after ankle arthrodesis is described in one comparative single center trial [56].

In addition, there are a large number of literature reviews and individual studies on unplanned reoperation rates after ankle replacement. A meta-analysis [36] including 17 studies reported rates similar to ours: in the > = 5-year follow-up, the unplanned reoperation rate was 29%, and in the > = 10-year follow-up, the unplanned reoperation rate was estimated at 42%. According to an evaluation of the German ankle replacement registry of the German Association for Foot and Ankle (D.A.F.), unplanned reoperations were documented in 13.5% of cases within a 2.5-year period between 2010 and 2012 [57]. These rates are consistent with the results of our event-time analyses.

Kaplan-Meier curves showed that unplanned reoperations after an ankle arthrodesis tend to occur at the beginning of the postoperative phase, whereas after total ankle replacement they are more evenly distributed over the postoperative period. These temporal patterns are not surprising, since loosening of the implant and material failure are to be expected only after a certain period of time [36]. Conversely, in ankle arthrodesis, complications associated with unplanned reoperations such as infections and pseudarthrosis usually rather occur at the beginning of the post-operative period. Furthermore, material failure after ankle arthrodesis is unlikely [58]; the only relevant late complication, apart from a rare secondary, hematogenous infection of the osteosynthesis material, is symptomatic subsequent arthrosis of the adjacent joints.

### Risk factors for unplanned reoperations

The risk factors identified as significant for the occurrence of unplanned reoperations after total ankle replacement (i.e., age < 50 and the presence of osteoporosis) correspond to the relative contraindications for ankle replacement already discussed in this paper and to the findings in literature reviews and clinical trials [1, 9]. With regard to the relative contraindication of increased BMI, the odds ratio of patients with obesity for an unplanned reoperation was 2.37, but the 95% CI [0.90;6.28] included 1. For patients with diabetes mellitus, no increased risk for an unplanned reoperation was found. However, with only 11 diagnosed diabetes mellitus cases, no valid conclusions can be drawn. The aspect of an earlier generation of implants as a risk factor for unplanned reoperations frequently mentioned in the literature [59, 60], in particular due to cemented fixation, cannot be discussed in this work, as all implants used belong to the third generation of implants.

The significant risk factors after ankle arthrodesis (i.e., higher overall patient-related severity, presence of osteoporosis, and presence of diabetes mellitus) were also consistent with the relevant literature. Indeed, it has been reported that the presence of diabetes mellitus and a higher American Society of Anesthesiologists grade increases the risk of complications after hindfoot arthrodesis, especially for failure of bone healing [61, 62]. A study of septic ankle arthrodesis with external fixator identified diabetes mellitus as a risk factor for complications, and the risk was also significantly increased for men [63]. According to Thevendran et al. [47], osteoporosis is also a risk factor for failure of bone healing.

Based on the odds ratios determined, the presence of obesity appears to result in a higher risk of an unplanned reoperation in both groups, though the association was not significant in this analysis. With regard to ankle arthrodesis, such an association is assumed in the literature [47], but after total ankle replacement, there are no consistent results regarding such a relationship [1, 64, 65]. Our results on the proportion of unplanned reoperations, stratified by osteoarthritis type, are supported by the relevant literature. Total ankle replacement is mainly recommended in less active older patients with primary osteoarthritis, while ankle arthrodesis is rather recommended in post-traumatic osteoarthritis due to the younger age of patients [9, 66]. Pyevich et al. [67] also showed that patients with post-traumatic osteoarthritis reported significantly more pain than patients with primary osteoarthritis after total ankle replacement with agility prostheses. Similarly, Popelka et al. [68] reported significantly lower American Orthopaedic Foot and Ankle Society (AOFAS) scores in patients with post-traumatic osteoarthritis than in patients with primary osteoarthritis after total ankle replacement. However, as the majority of patients in our analysis could not be assigned to an osteoarthritis type due to a lack of ICD specification, these results must be interpreted with caution.

### Limitations

One limitation of this work is the nature of a retrospective analysis of secondary data, in which additional parameters, such as postoperative pain levels or functional outcomes, cannot be collected in a targeted manner, but were collected for a different purpose.

Another limitation is that interventions after the initial surgery may be evaluated as follow-up interventions that have only a temporal but no causal relationship with the index surgery. In contrast to a prospectively planned study, special strategies are needed to deal with this issue in secondary data analyses [69]. To minimize the risk of bias, neither skin/subcutaneous surgeries more than 3 months after the index surgery were counted as unplanned reoperations, nor were osteosyntheses that were caused by a fracture or dislocation based on ICD coding.

Moreover, when identifying follow-up procedures in the present data set, two-stage total ankle replacement changes were counted as a total ankle replacement explantation due to the indistinguishable ICD codes. However, this does not effect the calculation of the incidence of unplanned reoperations remains unchanged. A bias of the incidence of unplanned reoperation rates can thus be excluded.

Furthermore, when identifying risk factors for unplanned reoperation rates in the logistic regression analysis, the number of cases in the group of total ankle replacement, with 172 patients, was significantly smaller than in the group of ankle arthrodesis, with 741 patients. Therefore, the possibility must be considered that with a larger number of cases, more of the investigated predictors would also have been detected as significant risk factors in the total ankle replacement group and vice versa.

## Conclusions

Similar unplanned reoperation rates and revision rates for both procedures, especially in the later study period, are likely due to a learning curve for arthroplasty surgeons and an improvement in implant designs. Based on this analysis of health insurance data in the 2001–2012 documentation period, an increase in implantations of total ankle replacements seems advisable. Moreover, a slight increase in reoperations following ankle arthrodesis surgery has been observed in patients over time. This should be discussed carefully with patients prior to performing the procedure. Future research projects need to analyze whether risk factors such as diabetes mellitus or higher PCCL severity also influence the outcome of patients receiving total ankle replacement and should directly compare ankle arthrodesis with total ankle replacement. This can be achieved through a multicenter, randomized, controlled trial design using patient-reported outcome measures (PROMs) and the inclusion of non-completers in the analysis or through a nationwide obligatory register for both procedures with PROMs recorded.

### Electronic supplementary material

Below is the link to the electronic supplementary material.

Supplementary Material 1

## Data Availability

Due restrictions apply to health insurance data analyzed during the current study, it is not possible to share these data publicly. The datasets analyzed during the current study are available from the corresponding author on reasonable request.

## Abbreviations

AOK: Allgemeine Ortskrankenkasse [engl.:provider of statutory medical insurance]
BMI: Body Mass Index
CI: Confidence Interval
ICD: International Statistical Classification of Diseases and Related Health Problems
DRG: Diagnosis Related Group
n: Number of Patients
OPS: Operationen- und Prozedurenschlüssel [engl.:operation and procedure code]
OR: Odds Ratio
p: p-value
PCCL: Patient Clinical Complexity Level
PROMs: Patient-Reported Outcome Measures
SD: Standard Deviation
