# Supplementary material for: Outcome after total ankle replacement or ankle arthrodesis in end-stage ankle osteoarthritis on the basis of german-wide data: a retrospective comparative study over 10 years
Source: BMC Musculoskelet Disord. 2024 Jun 25;25:492. doi: 10.1186/s12891-024-07612-w (PMC11197266; doi:10.1186/s12891-024-07612-w)
Supplement: Supplementary file 1 — Supplementary Material 1 [file 12891_2024_7612_MOESM1_ESM.docx]

**Appendix Table 1** Patients’ baseline characteristics – Late cohort (5-year FU)

| **Variable** | **Ankle**  **Arthrodesis**  **(N=1168)** | **Total Ankle**  **Replacement**  **(N=561)** |
| --- | --- | --- |
| Age, Average ± SD [Range] | 58.6± 14.1  [0-92] | 59.7 ± 12.2  [18-93] |
| Age Group, n (%) |  |  |
| 0-17 | 18 (1.5) | 0 |
| 18-49 | 289 (24.7) | 113 (20.1) |
| 50-69 | 588 (50.3) | 316 (56.3) |
| >=70 | 273 (23.4) | 132 (23.5) |
| Sex, n (%) |  |  |
| Female | 585 (50.1) | 289 (51.5) |
| Male | 583 (49.9) | 272 (48.5) |
| PCCL severity grade, n (%) |  |  |
| 0 | 622 (53.3) | 357 (63.6) |
| 1 | 1 (0.1) | 1 (0.2) |
| 2 | 146 (12.5) | 57 (10.2) |
| 3 | 198 (17.0) | 96 (17.1) |
| 4 | 201 (17.2) | 50 (8.9) |
| Diabetes mellitus, n (%) | 213 (18.2) | 63 (11.2) |
| Osteoporosis, n (%) | 193 (16.5) | 69 (12.3) |
| Obesity, n (%) | 228 (19.5) | 105 (18.7) |

Abbreviations: FU= Follow-Up, SD=Standard Deviation, n=Number of Patients, %=Proportion of Patients, PCCL=Patient Clinical Complexity Level

**Appendix Table 2** Number of patients with unplanned reoperations – stratified by age and gender

|  | **10-year FU**  **(N=913)** | | **5-year FU – Early cohort (N=913)** | | **5-year FU – Late cohort (N=1729)** | |
| --- | --- | --- | --- | --- | --- | --- |
| **Variable** | **Ankle**  **Arthrodesis**  **(N=741)** | **Total Ankle**  **Replacement (N=172)** | **Ankle**  **Arthrodesis**  **(N=741)** | **Total Ankle**  **Replacement (N=172)** | **Ankle**  **Arthrodesis**  **(N=1168)** | **Total Ankle**  **Replacement (N=561)** |
| Age, n (%) |  |  |  |  |  |  |
| 0-17 | 1 (5) | 0 | 1 (6) | 0 (0) | 5 (28) | 0 (0) |
| 18-49 | 43 (18) | 18 (46) | 37 (17) | 10 (29) | 67 (23) | 26 (23) |
| 50-69 | 84 (22) | 39 (37) | 67 (18) | 25 (23) | 127 (22) | 76 (24) |
| >=70 | 12 (13) | 8 (30) | 17 (13) | 8 (27) | 47 (17) | 28 (21) |
| Total | 140 (19) | 65 (38) | 122 (16) | 43 (25) | 246 (21) | 130 (23) |
| Sex, n (%) |  |  |  |  |  |  |
| Female | 62 (19) | 32 (33) | 54 (15) | 23 (23) | 114 (19) | 68 (24) |
| Male | 78 (19) | 33 (43) | 68 (17) | 20 (27) | 132 (23) | 62 (23) |
| Total | 140 (19) | 65 (38) | 122 (16) | 43 (25) | 246 (21) | 130 (23) |

Abbreviations: FU= Follow-Up, n=Number of Patients, %=Proportion of Patients

**Appendix Table 3** Number of patients with unplanned reoperations – By OA subtype (10-year FU)

| **Ankle Arthrodesis** | **Primary OA**  **(N=92), n(%)** | **Secondary OA**  **(N=118), n(%)** | **Posttraumatic OA (N=237), n(%)** |
| --- | --- | --- | --- |
| Rearthrodesis | 18 (20) | 12 (10) | 22 (9) |
| Osteosynthesis of bone | 11 (12) | 8 (7) | 12 (5) |
| Operations on skin and subcutaneous tissue | 7 (8) | 9 (8) | 12 (5) |
| Conversion to Endoprosthesis | 2 (2) | 2 (2) | 2 (1) |
| Sum of patients with unplanned reoperations | 25 (27)* | 24 (20) | 38 (16) |
| **Total Ankle Replacement** | **Primary OA**  **(N=42), n(%)** | **Secondary OA**  **(N=32), n(%)** | **Posttraumatic OA (N=47), n(%)** |
| Conversion to Arthrodesis | 5 (12) | 6 (19) | 7 (15) |
| Removal of Endoprosthesis | 6 (14) | 6 (19) | 4 (9) |
| Revision of Endoprosthesis or individual components | 2 (5) | 3 (9) | 7 (15) |
| Exposure of Endoprosthesis without replacement | 2 (5) | 2 (6) | 3 (6) |
| Operations on skin and subcutaneous tissue | 0 | 3 (9) | 5 (11) |
| Sum of patients with unplanned reoperations | 9 (21)* | 12 (37) | 14 (30) |

Abbreviations: OA=Osteoarthritis, FU= Follow-Up, n=Number of Patients, %=Proportion of Patients

*=Patients with different types of surgeries are only counted once in the sum - for this reason, the sum of patients does not correspond to the sum of number of patients in the subcategories
